# Vet-OncoNet: Developing a Network of Veterinary Oncology and Reporting a Pioneering Portuguese Experience

**DOI:** 10.3390/vetsci9020072

**Published:** 2022-02-07

**Authors:** Katia Pinello, Isabel Pires, Ana Filipa Castro, Paulo Tiago Carvalho, Andreia Santos, Augusto de Matos, Felisbina Queiroga, João Niza-Ribeiro

**Affiliations:** 1 Vet-OncoNet, Departamento de Estudo de Populações, ICBAS, Instituto de Ciências Biomédicas Abel Salazar, Universidade do Porto, Rua de Jorge Viterbo Ferreira 228, 4050-313 Porto, Portugal; up201505657@edu.icbas.up.pt (A.F.C.); up200703166@edu.icbas.up.pt (P.T.C.); jjribeiro@icbas.up.pt (J.N.-R.); 2 EPIUnit—Instituto de Saúde Pública, Universidade do Porto, Rua das Taipas 135, 4050-600 Porto, Portugal; 3 Laboratório para a Investigação Integrativa e Translacional em Saúde Populacional (ITR), Rua das Taipas 135, 4050-600 Porto, Portugal; 4 Departamento de Ciências Veterinárias, Universidade Trás-os-Montes e Alto Douro (UTAD), Quinta de Prados, 5000-801 Vila Real, Portugal; ipires@utad.pt (I.P.); fqueirog@utad.pt (F.Q.); 5 CECAV—Centro de Ciência Animal e Veterinária, Universidade de Trás-os-Montes e Alto Douro, Quinta de Prados, Apartado 1013, 5001-801 Vila Real, Portugal; 6 Departamento de Clínicas Veterinárias, ICBAS, Instituto de Ciências Biomédicas de Abel Salazar, Universidade do Porto, Rua de Jorge Viterbo Ferreira 228, 4050-313 Porto, Portugal; aasantos@icbas.up.pt (A.S.); ajmatos@icbas.up.pt (A.d.M.); 7 CECA-ICETA—Centro de Estudos de Ciência Animal, Instituto de Ciências, Tecnologias e Agroambiente, Universidade do Porto, 4051-401 Porto, Portugal

**Keywords:** cancer, comparative oncology, database, epidemiology, veterinary

## Abstract

Vet-OncoNet is a replicable tripartite animal cancer database with the scientific and academic purposes of collecting data and producing evidence-based knowledge for cancer science in general. Inspired by the One Health vision, Vet-OncoNet uses business intelligence tools to optimize the process of capturing, treating, and reporting animal cancer data to a national level in three interfaces: ACR (animal cancer registry, pathology-based), COR (clinical oncology registry, vet practice-based) and RFR (risk factor registry, owner-based). The first results show that skin and mammary gland are by far the most affected systems. Mast cell tumors and complex adenoma of the mammary gland are the most frequent histologic type in dogs, while in cats they are squamous cell carcinomas, tubular adenocarcinoma of the mammary gland and lymphomas. Regarding COR, it provides valuable information on the landscape of veterinary oncology practices, therapeutics options, outcomes and owners’ drivers’ adherence towards therapies, which range from 30% up to 80% upon vet practices. Furthermore, being aware of the role of animals within the family and as possible sentinels of environmental risks to cancer in humans, the network built an interface (Pet-OncoNet) dedicated to owners and a database (RFR) that receives information regarding pets and owners’ daily habits.

## 1. Introduction

The topic of oncology in companion animals is of growing clinical and epidemiological importance [1], with over 4.2 million dogs (approx. 5300/100,000 population rate) in the USA [2] and 412 out of 100,000 cats being diagnosed with cancer annually [3]. Common cancers in companion animals have been increasingly proposed as reliable and clinically relevant models of human disease [1,2] and the results acquired from companion animals with cancer would enable the scientific community to improve prevention strategies, diagnostic approaches, as well as the effectiveness and safety of new cancer therapy options for humans and for cancer-affected animal patients [1,4,5]. 

Accurate cancer surveillance data are part of the foundation needed to make appropriate conclusions about this burden of cancer, to understand the role of companion animals as sentinels of human neoplastic diseases [6,7,8,9], to set cancer strategies for prevention and control, and to design analytic studies to identify causal associations between exposures and cancer risk [10]. The first companion animal cancer registries were introduced in the 1960s [11,12], with several regional and country-level initiatives being developed over [13]. Unfortunately, many initiatives have not been continued for different reasons. Currently, few registry systems for animal oncology are operating at the global level. Fortunately, the era of Big Data has opened vast opportunities for launching initiatives in this domain, such as the Small Animal Veterinary Surveillance Network (SAVSNET) [14], the Veterinary Companion Animal Surveillance System (VetCompass) [15] and The Veterinary Medical DataBase [16].

This short communication aims to describe the Vet-OncoNet system, a Portuguese project inspired by the One Health vision and to report its existence. Vet-OncoNet focuses on animal cancer surveillance and is being developed with the ambition to produce evidence and knowledge not only on the veterinary oncology field, but also on comparative oncology, contributing to improve, in the medium and long term, both human and animal health.

## 2. The Vet-OncoNet project

Vet-OncoNet—The Veterinary Oncology Network [17], was officially launched in December 2019, an initiative held by ICBAS in partnership with Public Health Institute (ISPUP), University of Porto and Trás-os-Montes and Alto Douro University (UTAD). Vet-OncoNet’s mission is to produce scientific evidence and knowledge on animal oncology, bearing in mind the perspective of One Health, as well as to provide streamlined communication in animal oncology to veterinary clinics and pet owners. The project, initially driven by ICBAS, is an institutional initiative from the partners and was granted an internal grant intended to generate momentum. One permanent researcher was assigned, and intense networking and communication activities are key elements of daily routines. One of the core tasks is the creation of a system of registries on animal oncology, with particular emphasis on the Portuguese Animal Cancer Registry (ACR), similar in scope to those existing in the other countries [18,19,20,21,22,23]. Thus, some core activities of the Vet-OncoNet network are the collection, processing and analysis of data in databases dispersed across veterinary laboratories and veterinary clinical practices/hospitals.

**Figure 1:**
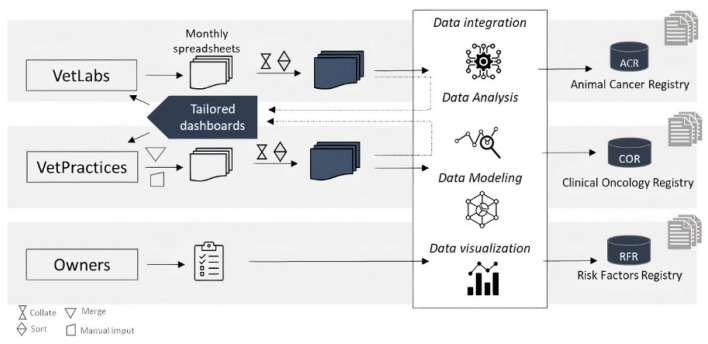
Illustration of the Vet-OncoNet data management system. Representation of the outputs of the system, structured data from Animal Cancer Registry, Clinical Oncology Registry and Risk Factors Registry, and the dashboards to partners.

The Vet-OncoNet developed its own information system (Figure 1), using SQL, R and business intelligence tools. The system sets on three databases designed with the objective of collecting information from different and complementary sources. The variables collected for each database are listed in Table 1.

### 2.1. Data Processing

After entering the system, the data undergoes a first stage of data cleaning and treatment that comprises editing, validation, standardization of the terms and classification (Figure 1). Each tumor record is classified accordingly to the final draft of Vet-ICD-O classification, which classifies the tumor into a topography and a histological type (morphology). This classification system is being developed by an international group—the Global Initiative on Veterinary Cancer Surveillance (GIVCS) [24], of which Vet-OncoNet members are included. This classification system is the canine counterpart of the human classification ICD-O-3.2, and it will allow comparability between veterinary and human cancer registries, supporting future comparative studies.

After the standardization of terms and classification of neoplasms, data is moved to the next step of epidemiological analysis and the interactive reports generation.

### 2.2. Data Delivery

Individualized interactive reports (dashboards) resulting from the treatment of data received, are an asset that all Vet-OncoNet partners can access permanently, via Web service (anytime, anywhere). These interactive reports allow each network partner to perform a dynamic visualization and analysis of their own data and a summarized real-time information.

## 3. Databases’ Preliminary Results

Vet-OncoNet has completed its first year of data recording in 2020. During that year, more than ten thousand neoplasms were reported from 6 VetLabs (70% of the Portuguese animal cancer diagnoses) and 27 VetPractices. Vet-OncoNet receives data from every animal group, however, the great majority of which comes from dogs (80.2%), with a higher proportion (60.0%) of females (Table 2).

### 3.1. Animal Cancer Registry

**Figure 2:**
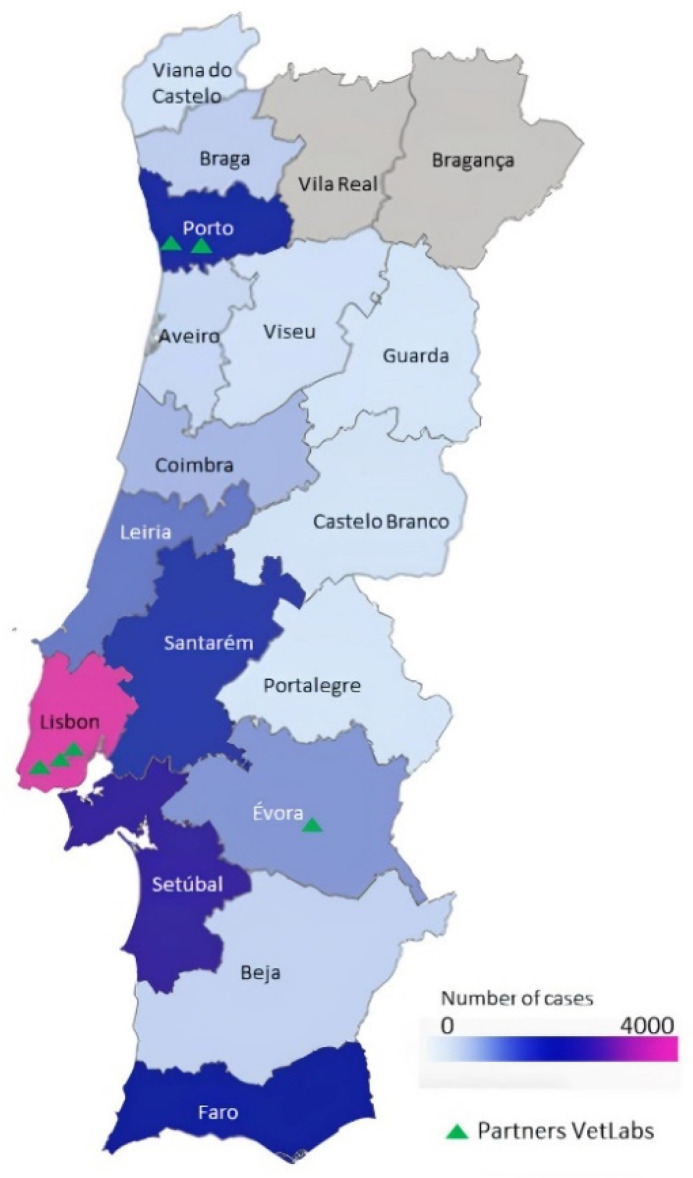
A choropleth map of animal cancer registries per districts, calculated based on postal code reported on Vet-OncoNet ACR. In Lisbon—DNATECH, VetPat^®^ and the Laboratory of Pathological Anatomy—Faculty of Veterinary Medicine, University of Lisbon; at Porto—the Laboratory of Veterinary Pathology, University of Porto and SEGALAB^®^; in Évora—the Laboratory of Veterinary Pathology, University of Évora.

The first database collects data from veterinary laboratories (VetLabs) producing the ACR [25]. Each registry entering Vet-OncoNet represents a confirmed animal cancer diagnosis and is regarded as a pathology-based registry. The VetLabs partners in 2020 were: In Lisbon—DNATECH, VetPat^®^ and the Laboratory of Pathological Anatomy—Faculty of Veterinary Medicine, University of Lisbon; at Porto—the Laboratory of Veterinary Pathology, University of Porto and SEGALAB; in Évora—the Laboratory of Veterinary Pathology, University of Évora (Figure 2). Registries are localized based on postal code reported.

The first results of Portuguese ACR can be consulted in the first edition of the Portuguese Animal Oncological Registry [25], which analyzed 8384 records from the database. After data analysis, results can be summarized as shown in Table 3.

### 3.2. Clinical Oncology Registry

The data from the veterinary clinical practices/hospitals (VetPractices) are collected into a second database, independent from ACR: The Clinical Oncological Registry. The COR registers clinical information such as proportion of cancer diagnostics in clinical practice, method of diagnostic and therapeutic more frequently used, cancer staging and outcome of cases.

The first results from COR show that cytology is the most frequent method of diagnosis (40.3%), followed by histopathology (35.6%) and in combination accounted for 11.5%. Even with the high variability and heterogeneity in clinical records between Vet Practices, and problems associated with the lack of information, two patterns could be disclosed. First, a predominance of surgical interventions over chemical-based therapies (40.4% and 17.4%). Second, a broad range in the adherence of animal owners to cancer therapies: from less than 30% to up 80%.

We consider the information coming to Vet-OncoNet from Vet Practices extremely important. This information allows understanding the landscape of veterinary oncology practices in the country. Only through this part of the system will it be possible to understand the methods of diagnosis, the staging procedures and its results, as well as treatments and the respective outcomes. Obtaining more solid evidence from Vet Practices could contribute to help veterinary oncology to progress to a new era of screening and prevention. Furthermore, it is important to understand the reasons driving owners and veterinarians’ decisions, e.g., to not undertaking or giving up therapy options, and to devise alternatives to increase access to cancer treatment in oncologic pets.

### 3.3. Risk Factors Registry

Vet-OncoNet created an interface to establish a communication channel to the society, with particular emphasis on owners of oncologic pets: Pet-OncoNet. The platform provides reliable information to help owners understand to better the disease in their pets, and the appropriate care to be provided. A collaboration with the Oncowaf initiative [26] was agreed to optimize efforts. Pet-OncoNet also provides a platform to collect data regarding cancer risk factors, from an online questionnaire available at the site. The RFR is a systematic collection of risk factors from owners with (case pets) and without cancer (control pets); it will collect extensive data from the entire country allowing us, in the future, to perform risk factors-based case–control studies.

## 4. Vet-OncoNet and Animal Census

Vet-OncoNet is a partner of the Portuguese Companion Animal Information System (SIAC), which is the Portuguese official site for compulsive registry of pets, providing the pet national census. Dogs, cats, and ferrets are the species included in the scope of SIAC. As a partner of SIAC since August 2021, Vet-OncoNet is responsible to perform the demographic treatment and analysis of the Portuguese pet population. The Portuguese pet census is available to Vet-OncoNet, after this partnership agreement, and allows calculating *population-based* cancer indicators. The adoption of the animal census in our calculations will be of utmost importance and an unprecedented achievement at the pet level. The use of the animal-based risk estimates will permit us to perform comparative studies of tumor risk incidence-based on human population and dog or cat cancer data.

The partnership agreement with SIAC is of utmost relevance because it will allow for the calculation of risk-based tumor incidence for pets, and it will permit comparisons between human and animal cancer occurrence

## 5. Conclusions

Animal cancer registries are a fundamental tool to produce evidence of the real occurrence and distributions of tumors in animals and should be progressively implemented across countries. Vet-OncoNet is a replicable tripartite animal cancer database aligned with the veterinary reality, using business intelligence tools to optimize the process of capturing, treating, and reporting animal cancer data.

Only with the participation, commitment, and work of all our partners—laboratories, Vet Practices, and owners—it was, and it will be, possible to create a data structure and a dimension that allows the generation of sound evidence, which would be impossible to produce with the current dispersed information.

## Figures and Tables

**Table 1:** Requested variables of each database of Vet-OncoNet.

Name of Data System	Data Source	Variables
Animal Cancer Registry (ACR)	Pathology reports from Veterinary Pathological Laboratories	Laboratory
		-Vet-OncoNet code
		Practice
		-Postal Code
		-City
		Tumor
		-Report ID
		-Date of diagnosis
		-Species
		-Sex
		-Breed
		-Age
		-Topography
		-Diagnosis
		-Grade
		-Method of diagnosis (histopathology, cytology, necropsy)
Clinical Oncology Registry (COR)	Data from Veterinary Practices, after oncology routine	Practice
		-Vet-OncoNet code
		Owner
		-Postal Code
		-City
		Animal
		-Species
		-Breed
		-Age
		-Sex
		Tumor
		-Topography
		-Diagnosis
		-Grade
		-Method of diagnosis
		-Treatment
		-Outcome
Risk Factors Registry (RFR)	Owners of oncologic patients	Questionnaire prepared to collect data from several risk factors from the animal, feeding habits, its environment, owners and family behavior.

**Table 2:** Summary statistics from the first year, 2020, of Vet-OncoNet.

Number of VetPractices	27
Number of laboratories	6 ^†^
Number of tumor registries	10,137
Number of animal groups	10 ^‡^
Proportion of dogs	80.2%
Proportion of cats	18.7%
Ratio cats: dogs	1:4.3
Ratio male: female	1:1.5

^†^ 6 LabVets out of 8 in Portugal. ^‡^ Canidae, Felidae, Leporidae, Rodentia (order), Equidae, Bovidae, Reptilia (class), Mustelidae, Birds (class), Fish (superclass).

**Table 3:** Main affected topographies and morphologies of dogs and cats from the first year of Vet-OncoNet [25].

Dogs (*n* = 6877)		Cats (*n* = 1624)	
Top 5 topographies	%		%
1. Skin 2. Mammary gland 3. Subcutaneous and soft tissue 4. Testis 5. Gum	50.9 21.9 7.3 4.2 3.3	1. Skin 2. Mammary gland 3. Digestive organs 4. Nasal Cavity and middle ear 5. Subcutaneous and soft tissue	38.7 35.5 6.1 3.1 3.0
Top 5 morphologies	%		%
1. Mast cell tumors 2. Lipoma 3. Complex adenoma ^1^ 4. Histiocytoma 5. Benign Mixed Tumor ^1^	9.4 5.5 4.7 3.7 3.7	1. Squamous cell carcinoma 2. Tubular adenocarcinoma ^1^ 3. Lymphomas 4. Tubule-papillary adenocarcinoma ^1^ 5. Solid carcinoma ^1^	11.5 11.4 6.9 6.5 6.5

^1^ Mammary gland.

## Data Availability

The data presented in this study are available on request from the corresponding author.
